# Pan-American Medical Congress, Havana, December 26, 27, 28 and 29, 1900

**Published:** 1900-12

**Authors:** 


### Pan-American Medical Congress, Havana, December 26, 27, 28 and 29, 1900.

The following announcement for the coming meeting of the Pan-American Congress, in Havana, December 26, 27, 28 and 29, 1900, has been received from the Secretary, Dr. Tomas V. Coronado, of Havana:

THE THIRD PAN-AMERICAN MEDICAL CONGRESS.

*Executive Committee.*—Dr. Juan Santos Fernandez, President; Dr. Gustavo Lopez, Vice-President; Dr. Enrique Acosta, Treasurer; Dr. Vincente B. Valdes, Dr. Jose Torralbas, Dr. Eduardo F. Pla, Committee Members; Tomas V. Coronado, Secretary.

HAVANA, CUBA, June, 1900.

*Dr. F. E. Daniel, Austin, Texas.*

DEAR SIR: As Secretary-General of the Executive Committee I have the honor to address you to ask for your co-operation in behalf of the third meeting of the Pan-American Congress.

Counting on your love for and devotion to professional matters, I would be extremely pleased if you would favor us with your personal assistance, and also by interesting your medical societies, universities and medical schools, as well as your prominent colleagues who are teaching the medical sciences so that they will take part in the Congress, which convenes on the 26th, 27th, 28th and 29th of December, and will send delegations, the members of which should forward their papers or titles according to the enclosed instructions.

The extraordinary impulse which experimentation and that multitude of auxiliary sciences whose progress amazes us has given to modern medicine affords the young American numerous advantages for study and investigations in the solution of many and varied problems that relate to the pathology and the pathogenesis of the numerous diseases.

Our climate, our soil, and the grade of civilization itself to which we have arrived produce appreciable modifications in the diseased conditions that develop in an atmosphere so distinct from that of Europe; and the diseases belonging to these altitudes, the study of which is being perfected each day, are sufficient motives to authorize the scientific investigations of our Congress. In these controversies the combined efforts do not represent the sum of the units that compose them, but represent the multiplication of them. The simultaneous work of all the investigations of the Americas presented and discussed at a given time surely must produce surprising results in reference to the utility and the practical application of the scientific productions of the men who cultivate the medical sciences on the American continent.

Each medical congress enables us to make progress in an effective way in the study of the laws of epidemiology, the preventive indications, the modifications that stamp the time, or the social surroundings in the medical topography of each territory, the quarantine, isolation, etc., in a word everything that relates to the progress of medical work up-to-date.

I sincerely hope that our sister nation, whose medical attainments are so famous, will co-operate in promoting the success of the Third Pan-American Congress by sending the largest possible representation, both in delegates and scientific productions.

I beg that you will mention in your reply the persons who will take part, and will send your papers at your earliest possible convenience.

Very truly yours,

TOMAS CORONADO.

Direction:

DR. TOMAS V. CORONADO,
Secretario de la Commission Ejecutiva del Tercer Congreso
Medico Pan-Americano,
Prado 105,
Habana, Cuba.

